# Elevated serum total immunoglobulin E is associated with an increased risk of lung cancer: a retrospective study

**DOI:** 10.3389/fimmu.2025.1637803

**Published:** 2025-07-21

**Authors:** Dongmei Zhou, Jia Liu, Chu Zhang, Dan Kang, Jiayang Kang, Zihan Meng, Xiaonan Wang

**Affiliations:** Department of Gerontology and Geriatrics, The First Hospital of China Medical University, Shenyang, Liaoning, China

**Keywords:** immunoglobulin E, lung cancer, clinical features, immunotherapy, biomarker

## Abstract

**Background:**

This study investigated the correlation between serum total IgE levels and the risk of lung cancer using univariate and multivariate logistic regression analysis.

**Methods:**

This cross-sectional retrospective cohort study included clinical and laboratory data from 675 lung cancer patients and 1,193 healthy controls.

**Results:**

The lung cancer patients showed significantly higher serum total IgE levels compared to healthy individuals, with 47.9% of patients having IgE levels >100 IU/ml (P < 0.01). Higher serum total IgE levels (>100 IU/ml) were significantly associated with increased risk of lung cancer (OR=1.534, 95% CI: 1.203-1.957; P < 0.001). Multivariate logistic regression analysis results showed that age ≥65 years (OR=4.775, 95% CI = 3.478–6.555; P <0.001), smoking history (OR=1.719, 95% CI = 1.198–2.466; P =0.003), and an elevated lymphocyte-to-monocyte ratio (LMR) (OR=0.777, 95% CI = 0.678–0.890; P <0.001) were independent risk factors for lung cancer development in subjects with serum total IgE levels >100 IU/ml. The higher the serum total IgE level, the higher the T stage, N stage, M stage and the later the tumor clinical stage (all P <0.001). Survival analysis did not show statistically significant differences in the median progression-free survival (PFS) (P>0.05) or overall survival (OS) (P>0.05) among advanced lung cancer patients with different IgE levels (high and low).

**Conclusion:**

Lung cancer patients demonstrated elevated serum total IgE levels. Higher serum IgE levels were significantly associated with an increased risk of lung cancer. Therefore, serum total IgE level is a potential diagnostic biomarker for lung cancer. Moreover, IgE and its related allergic immune responses offer potential as innovative therapeutic targets in elderly lung cancer patients with an history of smoking and elevated monocyte counts.

## 1 Introduction

Lung cancer is the leading cause of cancer-related deaths globally. In the last few decades, the incidence and mortality rates of lung cancer have shown an upward trend every year in China because of an aging population and unhealthy lifestyles. Lung cancer patients have several treatment options, including surgery, radiotherapy, chemotherapy, and targeted therapy. However, lack of pronounced early symptoms in most patients often lead to late-stage diagnoses (1). Therefore, there is an urgent need to identify new biomarkers for improving the early diagnosis rates and prognostic outcomes. Moreover, the immune system plays a key role in tumor development and progression. For example, immunoglobulin E (IgE) antibodies are not only important for mediating allergic reactions but also play a key role in regulating autoimmunity and the tumor microenvironment (2).

Several studies have reported that IgE is closely associated with the occurrence and development of various malignant tumors, especially in certain types of hematological malignancies and solid tumors (3). Low IgE levels are associated with an increased risk of certain types of cancer (such as hematologic malignancies), especially in high-risk populations exposed to carcinogens (4). In animal experiments, IgE-deficient mice exhibit accelerated tumor growth, thereby suggesting a key role for IgE in tumor immune surveillance (3). IgE also plays an important role in the tumor immune microenvironment and is associated with the progression of certain types of cancers (5). IgE promotes tumor cell apoptosis and inhibiting tumor growth through activation of the mast cells and eosinophils by binding to its high-affinity receptor, FcϵRI (6). Furthermore, the presence of IgE induces a shift in the tumor-associated macrophages (TAM) toward an anti-tumor phenotype, thereby enhancing immune surveillance against tumors (7). However, few studies have reported contradictory results and shown that IgE in the tumor microenvironment promotes polarization of macrophages into the M2 phenotype, which is associated with tumor progression and metastasis (8).

IgE also plays a significant role in the treatment of malignant tumors (8, 9). It exhibits high affinity for homologous Fcϵ receptors. This decreases the dissociation rate of IgE after receptor binding and increases the tissue residence time. This mechanistic detail has been used to develop novel cancer therapies involving IgE that are aimed at enhancing tumor targeting and immune activation (10–12). In lung cancer patients, serum IgE levels may reflect the biological behavior of the tumor and status of the immune system. Therefore, investigating the mechanistic role of IgE in lung cancer is not only important for gaining a deeper understanding of the tumor immune microenvironment but also characterizing novel therapeutic targets for clinical practice.

In this large-sample cross-sectional retrospective study, we investigated the relationship between serum total IgE levels and the risk of lung cancer. Furthermore, we evaluated the correlation between serum total IgE levels and various histologic types of lung cancer. We also analyzed the effects of serum total IgE levels on the prognosis of lung cancer patients. Overall, we aimed to develop a comprehensive understanding of the role of IgE in lung cancer to provide new insights for early screening and personalized intervention.

## 2 Methods

### 2.1 Patients

We included 675 cases diagnosed with lung cancer at the First Hospital of China Medical University between January 2014 and January 2024. The inclusion criteria was availability of definitive pathological results, clinical data, laboratory data, and baseline serum IgE levels prior to treatment. For comparison, we randomly selected 1,193 healthy control individuals who underwent serum IgE testing during the same period at the Medical Examination Center of the First Hospital of China Medical University. The control subjects did not have any history of malignant tumors (including solid tumors and pulmonary solid tumors) or severe chronic diseases. All procedures in this study were conducted in accordance with the Declaration of Helsinki guidelines (as revised in 2013). This study was approved by the Ethics Committee of the First Hospital of China Medical University (Project number: [2022] 371).

### 2.2 Data collection

The demographic and clinical characteristics were extracted from the electronic medical records of the patients, including age, gender, smoking history, alcohol consumption, medical history (hypertension or coronary heart disease or diabetes) and atopy-related diseases (asthma, eczema, or atopic dermatitis). We also extracted baseline peripheral blood data, including serum IgE level, absolute neutrophil count (ANC), absolute lymphocyte count (ALC), absolute eosinophil count (AEC), absolute monocyte count (AMC), c-reactive protein (CRP), interleukin-6 (IL-6), CD4+/CD8+ cell ratio, systemic immune-inflammation index ratio (SII: neutrophil count × platelet count)/lymphocyte count absolute monocyte count), neutrophil to lymphocyte ratio (NLR), platelet to lymphocyte Ratio (PLR), and lymphocyte to monocyte ratio (LMR). We also extracted clinical information of the lung cancer cases, including time of lung cancer diagnosis, pathological type, initial cancer stage, and prognosis. Progression-free survival (PFS) was defined as the time from the date of lung cancer diagnosis until disease progression. Overall survival (OS) was defined as the time from the date of lung cancer diagnosis until death or the last follow-up date.

### 2.3 Statistical analysis

All statistical tests were conducted using the SPSS 27.0 statistical software. P-value less than 0.05 was considered statistically significant. In the descriptive statistics section, categorical variables were represented as frequency counts or percentages (%). Differences between the case and control groups in the demographic variables and lifestyle factors were assessed using the chi-squared test. Continuous variables were represented as the median and interquartile range (IQR). Kruskal-Wallis test was used to evaluate intergroup differences for the clinical characteristics and blood parameters between lung cancer patients with different serum IgE levels. Unconditional logistic regression was used to analyze the relationship between serum IgE level and the overall risk of lung cancer or each pathological type of lung cancer (lung adenocarcinoma, lung squamous cell carcinoma, small cell lung cancer) after adjusting for, age, sex, smoking history, alcohol consumption, medical history, and atopy-related diseases, and the odds ratios (ORs) and 95% confidence intervals (95% CIs) were estimated. The association between clinical characteristics, blood parameters and the incidence of lung cancer in the population with elevated serum IgE level was analyzed using binary logistic regression analysis. Statistically significant indicators with P-values<0.05 were included in the multivariate logistic regression analysis. Subsequently, factors with P-values<0.05 in the multivariate analysis were considered as independent risk factors. Serum total IgE levels were compared between baseline and post treatment in advanced NSCLC and SCLC, using wilcoxon test. Kaplan-Meier survival curves were used to evaluate PFS and OS with 95% confidence intervals (CIs) of advanced lung cancer patients, and the log-rank test was used to assess whether the differences were statistically significant.

## 3 Results

### 3.1 Patient characteristics

This study included 675 lung cancer patients with baseline serum IgE levels after excluding 25 patients with unknown pathological types or incomplete clinical data. Furthermore, 1,193 healthy subjects who underwent total serum IgE testing were included as controls. The clinical characteristics of cases and controls are summarized in **Table 1**. There two groups did not show any statistically significant differences regarding the history of allergic diseases (P = 0.858). However, we observed significant differences in age, gender, smoking history, and total serum IgE levels between the two groups (all P<0.01). The lung cancer group also showed a higher percentage of individuals with smoking history compared to the control group (45.8% vs. 34.2%; P < 0.01). The male to female ratio in the control group was relatively balanced, but the proportion of male patients were significantly higher in the lung cancer group (68.4% vs. 31.2%).

**Table 1:** Baseline characteristics of control subjects and lung cancer patients.

Characteristics	Controls (n=1193)	LC (n=675)	P value
Age (years)			**<0.01**
<60	970 (0.813)	151(0.224)	
60-64	43 (0.036)	110 (0.163)	
65-69	72 (0.060)	180 (0.267)	
≥70	108 (0.091)	234 (0.346)	
Gender			**<0.01**
Female	615 (0.516)	213 (0.316)	
Male	578 (0.484)	462 (0.684)	
Smoking history			**<0.01**
Yes	408 (0.342)	309 (0.458)	
No	785 (0.658)	366 (0.542)	
Alcohol consumption			0.691
Nonregular	953 (0.799)	534 (0.791)	
Regular	240 (0.201)	141 (0.209)	
Medical History(Hypertension or Coronary heart disease or Diabetes )			0.155
Yes	465 (0.390)	240 (0.356)	
No	728 (0.610)	435 (0.644)	
Atopy-related diseases (Asthma or Eczema or Atopic dermatitis)			0.858
Yes	32 (0.026)	19 (0.028)	
No	1161 (0.973)	656 (0.972)	
Total Ig E (IU/ml)			**<0.01**
<25	314 (0.263)	144 (0.213)	
25-100	420 (0.352)	208 (0.308)	
>100	459 (0.385)	323 (0.479)	

Bold values indicate P < 0.01; N, number; LC, lung cancer.

Based on the serum total IgE levels, we classified both cases and controls into the following 3 groups: normal (<25 IU/mL), borderline (25–100 IU/mL), and elevated (>100 IU/mL). Compared to the controls, the lung cancer cases demonstrated statistically significant differences in the serum total IgE levels for all the three groups. Furthermore, the proportion of lung cancer cases in the serum total IgE >100 IU/ml subgroup were significantly higher than the controls (47.9% vs 38.5%; P <0.01).

### 3.2 Association between serum total IgE level and risk of lung cancer

**Figure 1:**
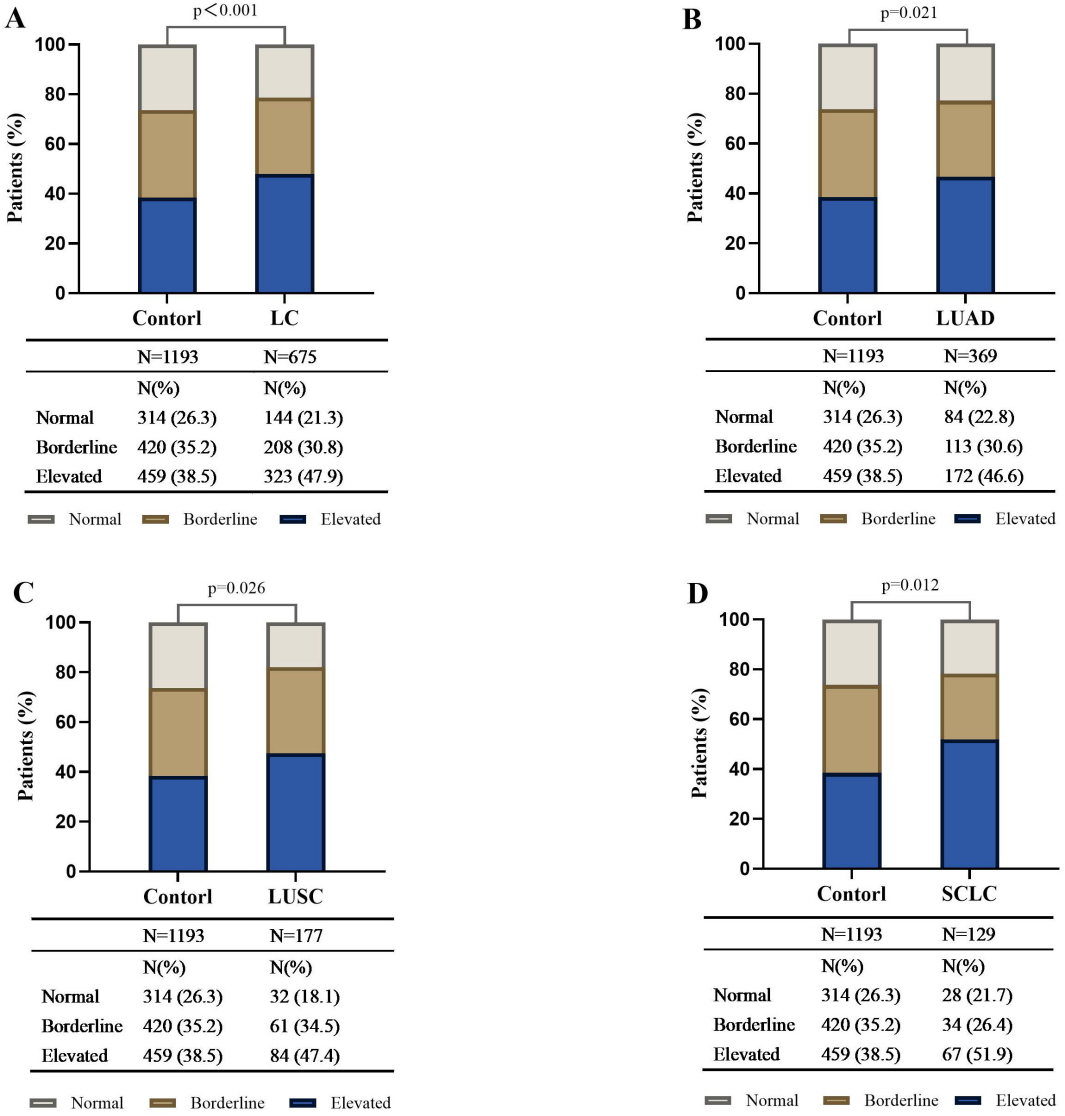
Distribution of different serum total IgE levels in control subjects, lung cancer patients, and different histologic types of lung cancer. The bar plot shows the proportion of subjects with IgE <25 IU/ml, 25≤IgE ≤ 100 IU/ml, and IgE >100 IU/ml in the healthy controls, lung cancer (LC) patients **(A)**, and LC patients in the LUAD **(B)**, LUSC **(C)**, and SCLC **(D)** histologic subgroups. Compared with the control, the distribution differences of serum total IgE among the three levels in the LC and by different histologic types were statistically significant and had a higher proportion in the IgE >100 IU/ml subgroup (all P <0.05). LC, lung cancer; SCLC, Small Cell Lung Cancer; NSCLC, non-small cell lung cancer; LUAD, lung adenocarcinoma; LUSC, lung squamous cell carcinoma.

The logistic regression analysis results showed that elevated serum total IgE levels were significantly associated with an increased risk of lung cancer after adjusting for age, sex, smoking history, alcohol consumption, medical history, and atopy-related diseases (OR 1.534, 95% CI 1.203-1.957; P < 0.001) (**Table 2**). Subsequently, lung cancer patients were categorized into different pathological types such as lung adenocarcinoma (LUAD), lung squamous cell carcinoma (LUSC), and small cell lung cancer (SCLC), and correlation of the pathological subtypes with the serum total IgE levels was evaluated. Our results demonstrated that the proportion of LUAD, LUSC and SCLC in the serum total IgE >100 IU/ml subgroup were significantly higher than the controls (all P<0.05) (**Figure 1**). As shown in **Table 2**, the OR value was 1.425 in the 25 ≤ IgE ≤ 100 IU/ml group LUSC patients. However, the IgE > 100 IU/ml group was associated with significantly higher risk for all the pathological types of lung cancer compared to the IgE <25 IU/ml group (all P<0.05).

**Table 2:** Association between serum total IgE level and risk of lung cancer and by different histologic types.

Total IgE status (IU/ml)	Controls N=1193 N (%)	LC N=675 N (%)	OR (95% CI) ^a^	LUAD N=369 N (%)	OR (95% CI) ^a^	LUSC N=177 N (%)	OR (95% CI) ^a^	SCLC N=129 N (%)	OR (95% CI) ^a^
IgE<25	314 (26.3)	144 (21.3)	1 (Ref)	84 (22.8)	1 (Ref)	32 (18.0)	1 (Ref)	28 (21.7)	1 (Ref)
25≤IgE ≤ 100	420 (35.2)	208 (30.8)	1.080 (0.834-1.398)	113 (30.6)	1.006 (0.732-1.382)	61 (34.5)	1.425 (0.907-2.224)	34 (26.4)	0.908 (0.539-1.529)
IgE>100	459 (38.5)	323 (47.9)	1.534 (1.203-1.957) ^*^	172 (46.6)	1.401 (1.040-1.887) ^*^	84 (47.5)	1.796 (1.166-2.765) ^*^	67 (51.9)	1.637 (1.029-2.603) ^*^

N, number; CI, confidence interval; OR, odds ratio; LC, lung cancer; LUAD, lung adenocarcinoma; LUSC, lung squamous cell carcinoma; SCLC, Small Cell Lung Cancer.

a OR and 95% CI were calculated using unconditional logistic regression, adjusted for, age, sex, smoking history, alcohol consumption, medical history, and atopy-related diseases. ^*^P<0.05

### 3.3 Clinical characteristics and laboratory findings of lung cancer patients in groups with different serum total IgE levels

Lung cancer patients were divided into three groups based on their baseline serum total IgE levels at diagnosis. As shown in **Table 3**, a higher proportion of patients in the IgE >100 IU/ml and 25≤IgE ≤ 100 IU/ml groups were males and aged ≥65 years. Moreover, patients in the smoking and alcohol consumption groups showed higher serum total IgE levels. Previous studies have shown that both smoking and alcohol consumption are associated with elevated serum IgE levels (13, 14). The ANC were significantly higher in the 25≤IgE ≤ 100 IU/ml and IgE>100 IU/ml groups compared to the IgE<25 IU/ml group. The AEC and AMC were significantly higher in the IgE>100 IU/ml group compared to the other two groups. This resulted in a lower LMR in the IgE>100 IU/ml group compared to the other two groups. However, there were no statistically significant differences in the atopy-related diseases, baseline ALC, NLR, PLR, CRP, IL-6, SII, and CD4+/CD8+ cell ratio between the 3 groups.

**Table 3:** Clinical features of lung cancer patients for clinical cut-offs of total IgE level.

Variables	IgE<25 IU/ml (n=144)	25≤IgE ≤ 100 IU/ml (n=208)	IgE>100 IU/ml (n=323)	P value
Age (years)				
<65	68 (47.2)	71 (34.1)	122 (37.8)	**0.042**
≥65	76 (52.8)	137 (65.9)	201 (62.2)	
Gender				
Female	72 (50.0)	71 (34.1)	70 (21.7)	**<0.01**
Male	72 (50.0)	137 (65.9)	253 (78.3)	
Smoking history				
Yes	46 (31.9)	93 (44.7)	170 (52.6)	**<0.01**
No	98 (68.1)	115 (55.3)	153 (47.4)	
Alcohol consumption				
Nonregular	128 (88.9)	171 (82.2)	235 (72.8)	**<0.01**
Regular	16 (11.1)	37 (17.8)	88 (27.2)	
Atopy-related diseases (Asthma or Eczema or Atopic dermatitis)				
Yes	3 (2.1)	4 (1.9)	12 (3.7)	0.394
No	141 (97.9)	204 (98.1)	311 (96.3)	
ANC (×10^9^/L)	3.86 (2.59-5.19)	4.51 (3.07-6.42)	4.34 (2.95-6.20)	**0.027**
ALC (×10^9^/L)	1.28 (0.923-1.83)	1.33 (1.00-1.83)	1.46 (1.02-1.87)	0.531
AEC (×10^9^/L)	0.09 (0.03-0.18)	0.11 (0.04-0.22)	0.13 (0.05-0.23)	**0.010**
AMC (×10^9^/L)	0.46 (0.35-0.67)	0.54 (0.41-0.75)	0.55 (0.42-0.74)	**0.004**
SII	612.43 (367.70-1142.00)	752.13 (430.65-1464.58)	704.06 (395.27-1306.96)	0.097
NLR	2.76 (1.59-5.04)	3.21 (2.03-5.33)	3.01 (1.91-5.17)	0.192
PLR	148.77 (121.04-245.12)	174.03 (122.32-254.43)	164.68 (115.91-247.21)	0.383
LMR	3.03 (1.81-4.35)	2.44 (1.77-3.58)	2.47 (1.52-3.68)	**0.047**
CRP (mg/L)	7.55 (3.21-41.10)	12.95 (4.00-52.50)	14.20 (4.00-56.90)	0.123
IL-6 (pg/ml)	8.68 (3.74-20.87)	14.44 (4.02-39.25)	12.8 (5.29-25.00)	0.181
CD4/CD8	1.44 (0.99-2.36)	1.48 (1.06-1.99)	1.45 (1.02-2.18)	0.996

Bold values indicate P < 0.05 that is considered statistically significant; ANC, absolute neutrophil count; ALC, absolute lymphocyte count; AEC, absolute eosinophil count; AMC, absolute monocyte count; SII, systemic immuneinflammation index ratio; NLR, neutrophil to lymphocyte ratio; PLR, platelet to lymphocyte Ratio; LMR, lymphocyte to monocyte ratio; CRP, c-reaction protein; IL-6, interleukin-6.

### 3.4 Relationship between IgE levels and lung cancer types and stages

This study further investigated the correlation between varying serum total IgE levels and the pathological classification as well as TNM staging of lung cancer. All patients diagnosed with NSCLC and SCLC were restaged according to the AJCC 8th edition TNM staging criteria. The results presented in **Table 4** indicated that there were no significant differences in the distribution of pathological types among patients with different serum total IgE levels. However, statistically significant differences were observed among the three groups in the distribution of T (primary tumor), N (regional lymph node involvement), M (distant metastasis), and TNM (pathological staging) (p < 0.001). Notably, as IgE levels increased, the proportion of higher-stage T, N, and M classifications exhibited a gradual upward trend.

**Table 4:** Relationship between elevated total IgE levels and pathological features of LC.

Variables	IgE<25 IU/ml (n=144)	25≤IgE ≤ 100 IU/ml (n=208)	IgE>100 IU/ml (n=323)	P-value
Histologic type				0.491
LUAD	84 (58.3)	113 (54.4)	172 (53.3)	
LUSC	32 (22.2)	61 (29.3)	84 (26.0)	
SCLC	28 (19.5)	34 (16.3)	67 (20.7)	
T stage				**<0.001**
T1	59 (42.8)	67 (33.4)	71 (22.6)	
T2	44 (31.9)	64 (31.8)	69 (22.0)	
T3	16 (11.6)	37 (18.4)	55 (17.5)	
T4	19 (13.7)	33 (16.4)	119 (37.9)	
N stage				**<0.001**
N0	55 (39.3)	75 (37.3)	39 (12.5)	
N1	20 (14.3)	21 (10.4)	12 (3.8)	
N2	54 (38.6)	60 (29.9)	84 (26.8)	
N3	11 (7.8)	45 (22.4)	178 (56.9)	
M stage				**<0.001**
M0	78 (56.9)	127 (63.2)	140 (44.7)	
M1	59 (43.1)	74 (36.8)	173 (55.3)	
CTNM				**<0.001**
I-II	49 (34.5)	78 (38.8)	46 (14.6)	
III	33 (23.2)	48 (23.9)	95 (39.3)	
IV	60 (42.2)	75 (37.3)	173 (55.1)	

Bold values indicate P < 0.05; LC, lung cancer; LUAD, lung adenocarcinoma; LUSC, lung squamous cell carcinoma; SCLC, Small Cell Lung Cancer; CTNM, clinical tumor node metastasis.

### 3.5 Changes in serum total IgE levels after treatment

**Figure 2:**
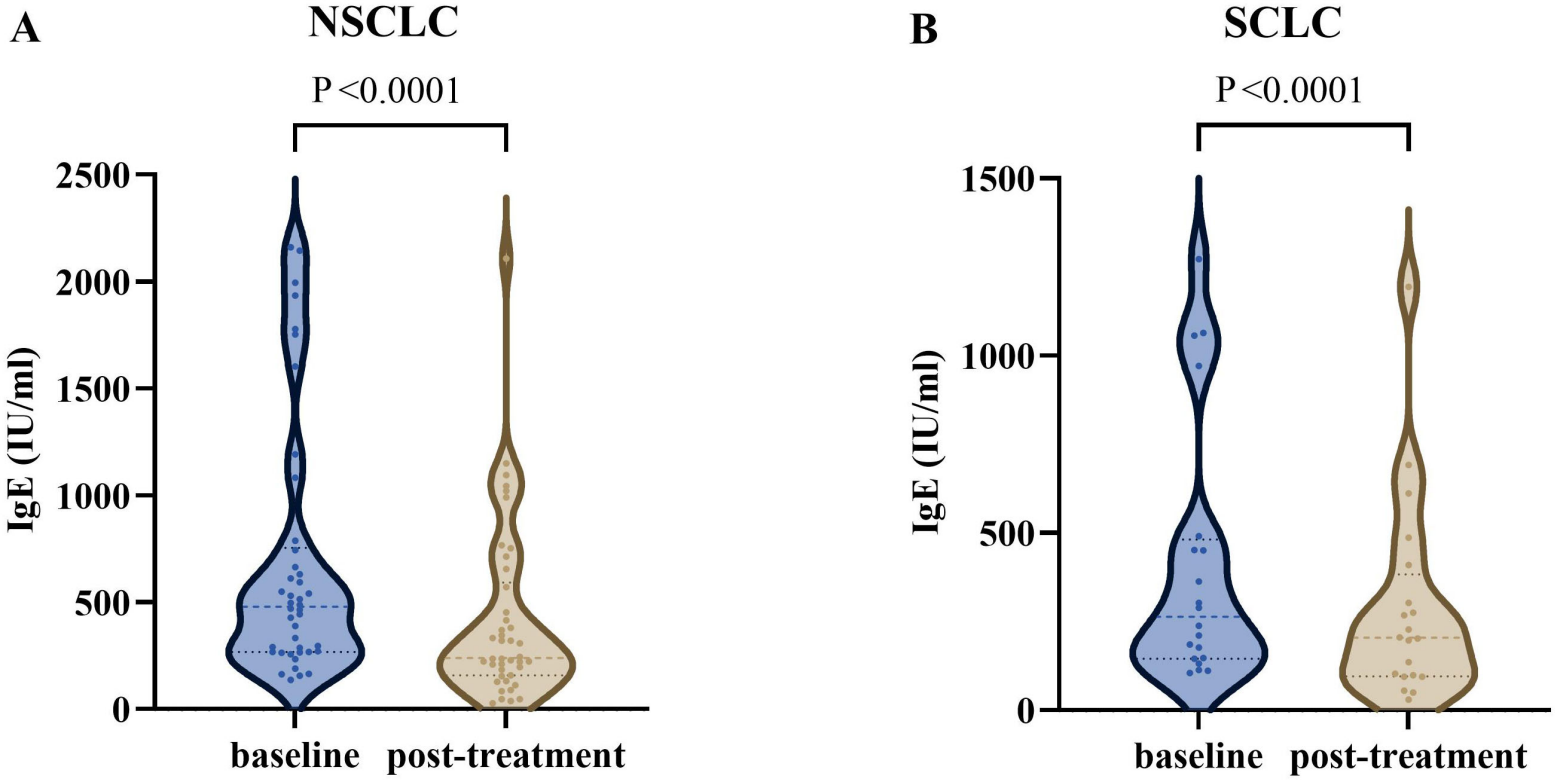
The serum total IgE levels in advanced lung cancer patients at different times. **(A)** The IgE level was significantly decreased after treatment in advanced NSCLC (P <0.0001). **(B)** The IgE level was significantly decreased after treatment in SCLC (P <0.0001). NSCLC, non-small cell lung cancer; SCLC, Small Cell Lung Cancer.

After excluding cases lacking post-treatment IgE test data, the final analysis included 42 advanced NSCLC patients and 20 SCLC patients. The median serum total IgE level in NSCLC patients significantly decreased from the baseline value of 479.0 IU/ml (IQR: 267.0–754.3) to 239.7 IU/ml (IQR: 157.2–592.0) post-treatment, with a median reduction of 239.3 IU/ml (IQR: 479.0–239.7; Z = -5.133, p < 0.001). Similarly, SCLC patients also exhibited a significant decline in IgE levels after treatment, with the baseline median of 263.8 IU/ml (IQR: 145.5–481.3) decreasing to 204.1 IU/ml (IQR: 96.0–382.9) post-treatment, resulting in a median reduction of 59.7 IU/ml (IQR: 263.8–204.1; Z = -3.823, p < 0.001) (**Figure 2**).

### 3.6 Associations between baseline risk factors and lung cancer in patients with elevated serum total IgE level

During the study period, we included 782 subjects (containing 459 healthy controls and 323 lung cancer patients) with IgE >100 IU/ml. Univariate logistic regression analysis demonstrated that factors such as age, gender, smoking history, ANC, AMC, NLR, PLR, CRP, ALC, AEC, LMR, and CD4+/CD8+ ratio were significantly associated with an increased risk of lung cancer in patients with IgE >100 IU/ml (**Table 5**). Subsequent multivariate logistic regression analysis demonstrated that age ≥65 years, history of smoking, lower LMR, and lower CD4+/CD8+ ratio were independent risk factors for the occurrence of lung cancer occurrence in patients with IgE >100 IU/ml.

**Table 5:** Univariate and multivariate logistic regression analysis for the risk factors of lung cancer in patients with elevated serum total IgE level.

Variables	Univariate analysis		Multivariate analysis	
	OR (95% CI)	P-value	OR (95% CI)	P-value
Age (years)				
<65	1 (Ref)	**<0.001**	1 (Ref)	**<0.001**
≥ 65	8.927 (7.152-11.142)		4.775 (3.478-6.555)	
Gender				
Female	1 (Ref)	**<0.001**	1 (Ref)	0.860
Male	2.308 (1.893-2.813)		0.968 (0.674-1.391)	
Smoking history				
No	1 (Ref)	**<0.001**	1 (Ref)	**0.003**
Yes	1.624 (1.339-1.970)		1.719 (1.198-2.466)	
Alcohol consumption				
Nonregular	1 (Ref)	0.691		
Regular	1.048 (0.830-1.324)			
Atopy-related diseases (Asthma or Eczema or Atopic dermatitis)				
No	1 (Ref)	0.809		
Yes	0.931 (0.524-1.657			
ANC (×10^9^/L)	1.042 (1.013-1.071)	**0.004**	1.000 (0.932-1.073)	0.993
ALC (×10^9^/L)	0.705 (0.621-0.800)	**<0.001**	1.049 (0.732-1.503)	0.794
AEC (×10^9^/L)	0.438 (0.274-0.698)	**<0.001**	0.740 (0.407-1.347)	0.325
AMC (×10^9^/L)	3.018 (2.096-4.345)	**<0.001**	0.703 (0.291-1.702)	0.435
SII	1.000 (1.000-1.001)	**<0.001**	1.000 (1.000-1.000)	0.294
NLR	1.082 (1.054-1.111)	**<0.001**	0.978 (0.938-1.020)	0.297
PLR	1.004 (1.003-1.005)	**<0.001**	1.000 (0.997-1.002)	0.730
LMR	0.816 (0.776-0.857)	**<0.001**	0.777 (0.678-0.890)	**<0.001**
CRP (mg/L)	1.008 (1.005-1.010)	**<0.001**	1.000 (0.997-003)	0.921
IL-6 (pg/ml)	1.003 (0.999-1.010)	0.078		
CD4/CD8	0.594 (0.484-0.624)	**<0.001**	0.582 (0.503-0.674)	**<0.001**

Bold values indicate P < 0.05; ANC, absolute neutrophil count; ALC, absolute lymphocyte count; AEC, absolute eosinophil count; AMC, absolute monocyte count; SII, systemic immuneinflammation index ratio; NLR, neutrophil to lymphocyte ratio; PLR, platelet to lymphocyte Ratio; LMR, lymphocyte to monocyte ratio; CRP, c-reaction protein; IL-6, interleukin-6.

In the IgE >100 IU/ml group of lung cancer patients, the risk of developing lung cancer in subjects aged ≥65 years was 4.775-fold higher than those aged <65 years (OR = 4.775, 95% CI = 3.478–6.555; P <0.001). Furthermore, the risk of lung cancer in patients with a smoking history was 1.719-fold higher compared to non-smokers (OR = 1.719, 95% CI = 1.198–2.466; P =0.003). The risk of lung cancer decreased by 22.3% for every 1-unit increase in LMR (OR = 0.777, 95% CI = 0.678–0.890; P <0.001) and by 41.8% for every 1-unit increase in the CD4+/CD8+ ratio (OR 0.582, 95% CI 0.503–0.674; P <0.001).

### 3.7 Association between serum total IgE level and clinical outcomes in advanced lung cancer patients

Since long-term clinical follow-up data was lacking for patients with stage I and II lung cancer and there was significant variability in the treatment of patients with stage III NSCLC, we could not analyze the association between serum total IgE level and clinical prognosis for these groups of patients. Therefore, we analyzed the follow-up data of 185 stage IV NSCLC patients and 59 extensive-stage small cell lung cancer (ES-SCLC) patients.

**Figure 3:**
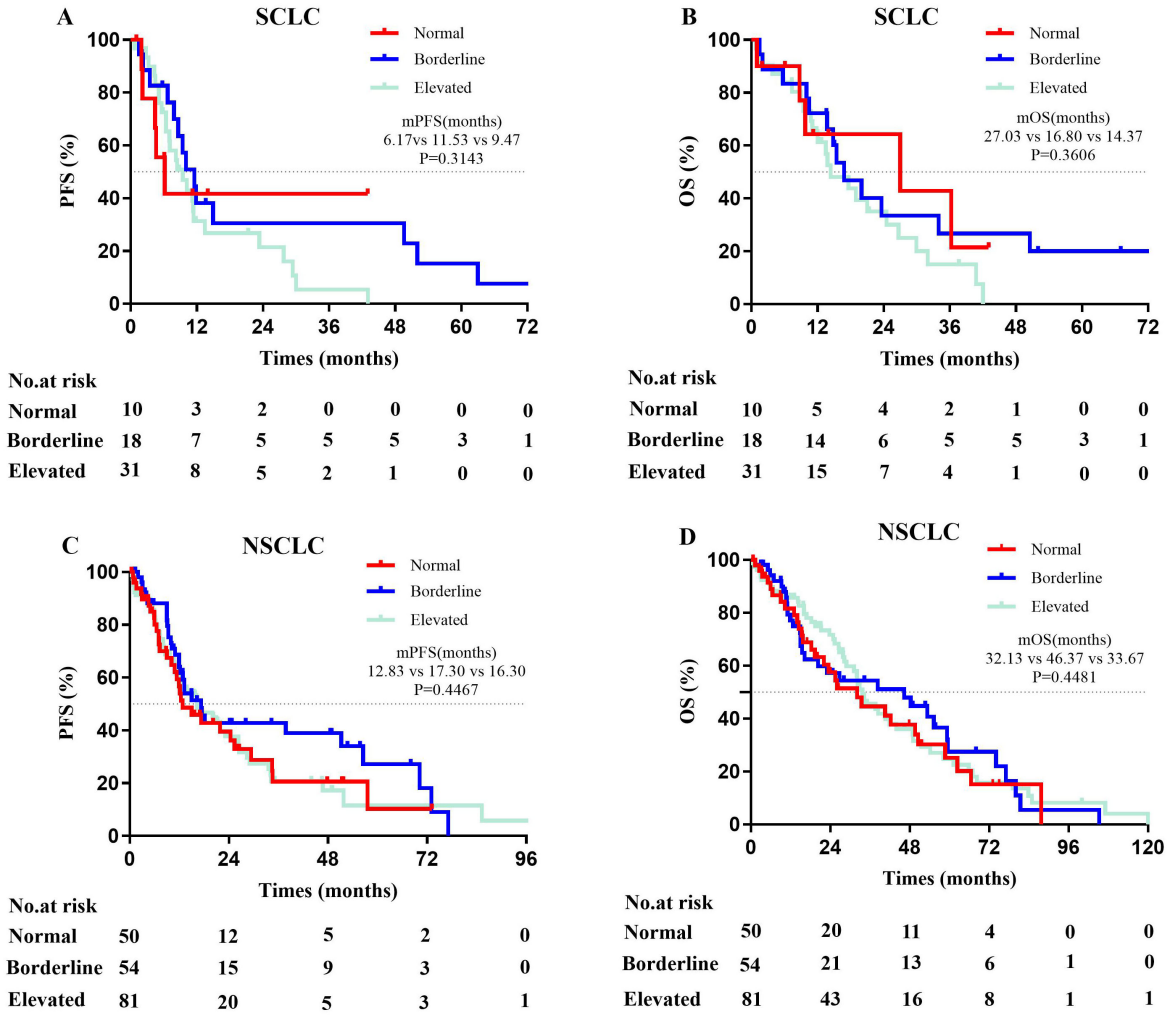
Kaplan-Meier curves for PFS and OS in advanced lung cancer patients. **(A, B)** The mPFS (χ2 = 2.315, p = 0.3143) and mOS (χ2 = 2.040, p=0.3606) among ES-SCLC patients (n=59) with varying serum total IgE levels were not statistically significant. **(C, D)** There were not significant difference in mPFS (χ2 = 1.612, p = 0.4467) and mOS (χ2 = 0.7993, p = 0.4481) among Stage IV NSCLC patients (n=185) with varying serum total IgE levels. Normal (Ig E<25 IU/mL); Borderline (25≤IgE ≤ 100 IU/ml); Elevated (IgE>100 IU/mL); PFS, progression-free survival; OS, overall survival; ES-SCLC, extensive-stage small cell lung cancer; NSCLC, non-small cell lung cancer.

Among ES-SCLC patients, those with normal serum total IgE levels showed the lowest median PFS (normal, 6.17 months; borderline, 11.53 months; elevated, 9.47 months; P=0.3143), whereas those with elevated serum total IgE level showed the lowest median OS (normal, 27.03 months; borderline, 16.80 months; elevated, 14.37 months; P=0.3606), but the results were not statistically significant (**Figures 3A, B**).

Among stage IV NSCLC patients, those with normal serum total IgE level showed the lowest median PFS (normal, 12.83 months; borderline, 17.30 months; elevated, 16.30 months; P=0.4467) and the lowest median OS (normal, 32.13 months; borderline, 46.37 months; elevated, 33.67 months; P=0.4481), but the results were not statistically significant (**Figures 3C, D**).

These results suggested that serum total IgE levels did not show a significant impact on the median PFS and OS of advanced lung cancer patients.

## 4 Discussion

This large-cohort retrospective study provides the first direct evidence of the relationship between the serum total IgE levels and lung cancer. Our findings demonstrated that serum total IgE levels were significantly higher in the lung cancer patients than in the healthy controls, and 47.9% of patients exhibited IgE levels exceeding 100 IU/ml (P < 0.01). Although previous studies have investigated the role of IgE in various tumor types, conclusive evidence regarding its correlation with lung cancer development and progression is lacking (15, 16). This study showed significant association between serum total IgE levels and lung cancer risk. In subjects with serum total IgE levels > 100 IU/ml, the risk of lung cancer was 1.534 times higher than in those with IgE levels < 25 IU/ml. A previous meta-analysis demonstrated that the risk of lung cancer was 1.8-fold higher in non-smoking asthma patients compared to non-smoking non-asthma patients (17). However, a systematic investigation conducted by the International Lung Cancer Consortium did not find a direct causal relationship between asthma and lung cancer risk (18). In this study, the proportion of control group subjects and lung cancer patients with an history of allergic diseases were comparable and did not show statistically significant differences (P = 0.858). Previous studies have shown that the relationship between malignancies and serum IgE levels are age-related (19, 20), with a higher proportion of lung cancer cases in subjects aged ≥70 years (34.6%) compared to those below 70 years. Smoking is another critical risk factor in the occurrence and progression of lung cancer (21), especially in China because of a large smoking population (22). Furthermore, multiple analyses have shown that males are more frequently diagnosed with lung cancer because of higher smoking rates (23). In this study, we demonstrated that serum total IgE levels correlated with age, gender, and history of smoking and alcohol consumption. These findings aligned accurately with previously reported findings about factors influencing serum total IgE levels.

The findings of this study indicate that there is no significant correlation between IgE levels and the pathological types of lung cancer. However, a significant positive correlation was observed between IgE levels and the TNM staging of tumors, meaning that higher TNM stages were associated with elevated total IgE levels. Following antitumor treatment, patients exhibited a downward trend in IgE levels, though further validation of this finding requires the accumulation of additional follow-up data. Prognostic analysis suggested that the survival outcomes in advanced lung cancer patients across various serum IgE levels were statistically insignificant. However, as shown in **Figure 3**, this was probably due to insufficient sample size. Consequently, to determine the prognostic role of serum IgE in lung cancer, larger sample sizes are necessary for the subgroup analyses based on different pathological types, stages, treatment regimens, and serum IgE levels. A recent clinical research study reported that elevated serum IgE levels are associated with improved prognosis in IDH wild-type gliomas (24). However, findings from lung cancer prognosis studies are inconsistent. Allergic reactions are inherently heterogeneous and exhibit diverse phenotypes, omics profiles, and pathophysiological processes (25). This may contribute to the variability observed in clinical research. This study also investigated the correlation between allergy history and the occurrence of lung cancer but did not find statistically significant differences while comparing lung cancer patients with healthy control subjects or different IgE level subgroups of lung cancer patients. Laboratory results also showed that higher serum IgE levels and a lower lymphocyte-to-monocyte ratio (LMR), rather than eosinophils, correlated with elevated risk of lung cancer. This suggests that IgE may play a significant role in the immune surveillance mechanisms or facilitate a tumor-promoting microenvironment in lung cancer. However, further research is necessary to determine the exact mechanistic role played by IgE in lung cancer occurrence and progression.

This study also showed that subjects with IgE >100 IU/ml were at a higher risk of lung cancer if they were also associated with additional risk factors such as elderly, smoking history, decreased lymphocyte-to-monocyte ratio (LMR), and immunosuppression of the lymphatic system. The LMR is a relatively new inflammation-related score and demonstrates a prognostic relationship with lung cancer outcomes (26). The biological mechanisms underlying this relationship of LMR with lung cancer outcomes remain unclear and cannot be solely explained by the functions of lymphocytes and monocytes in cancer. Lymphocytes play dual and contradictory roles in cancer. They can drive antitumor immune effects, but also promote metastasis by influencing the tumor microenvironment (27). On the other hand, monocytes promote tumor invasion and progression by differentiating into tumor-associated macrophages (TAM) (28). Therefore, the LMR index indicates the balance between lymphocytes and monocytes. This study suggests that elevated serum total IgE levels are linked to increased monocyte counts, thereby suggesting that the LMR reflects the effect of allergic immunity on the balance between lymphocytes and monocytes. This may also be related to the expression of FcϵR on the lymphocytes and monocytes (29, 30). The high-affinity IgE receptor FcϵRI is expressed on the mast cells, monocytes, macrophages, dendritic cells, and eosinophils. Recombinant IgE anticancer antibodies have been used to exert immune surveillance effects by exploiting their high affinity for the tumor immune effector cells (31). In a Phase I clinical study, MOv18 IgE antibody therapy showed significant antitumor activity in the ovarian cancer patients (10). This provides clinical evidence for the application of IgE-based immunotherapy in specific populations with lung cancer.

The findings of this study have multifaceted clinical significance. Firstly, this study demonstrated that serum total IgE levels, age, smoking history, and LMR are valuable lung cancer risk indicators that can be used by clinicians to more effectively screen high-risk populations, thereby improving early diagnosis. Furthermore, serum IgE, a hallmark biomarker of allergic immunity, was associated with lung cancer. Therefore, in-depth investigations are necessary to elucidate the mechanistic role of IgE in lung cancer pathogenesis. Most importantly, IgE-targeted therapies may offer novel treatment strategies for distinct subgroups of lung cancer patients such as elderly subjects with high IgE levels, a history of smoking, low LMR values, and lymphocyte immunodeficiency.

However, this study also has several limitations. Firstly, this was a retrospective study without a long-term follow-up. Therefore, we could not assess the causal relationship between serum total IgE levels and lung cancer risk. Secondly, although this study was conducted with a large sample size, we cannot generalize the findings because biological experimental validation and long-term follow-up was not conducted. Therefore, large-scale multi-center prospective studies should be conducted in the future with diverse populations to overcome batch-to-batch variations in the relationship between IgE levels and lung cancer risk. Moreover, the future multicenter longitudinal studies should also investigate specific mechanisms by which IgE contributes to lung cancer initiation and progression. Such studies will offer a more solid foundation for the early diagnosis and personalized treatment of lung cancer.

## 5 Conclusions

This is the first study to demonstrate a significant association between serum total IgE levels and the risk of lung cancer. The higher the serum total IgE level, the higher the T stage, N stage, M stage and the later the tumor clinical stage. Age ≥65 years, history of smoking, elevated LMR, and an elevated CD4+/CD8+ ratio are independent risk factors of lung cancer. The prognostic role of IgE in lung cancer requires further investigation. Nevertheless, therapeutic interventions targeting IgE and its related immune functions are promising novel treatment targets for lung cancer.

## Data Availability

The original contributions presented in the study are included in the article/supplementary material. Further inquiries can be directed to the corresponding author.

## References

[1] Miller KD Nogueira L Mariotto AB Rowland JH Yabroff KR Alfano CM. Cancer treatment and survivorship statistics, 2019. CA Cancer J Clin. (2019) 69:363–85. doi: 10.3322/caac.21565, PMID: 31184787

[2] Luker AJ Lownik JC Conrad DH Martin RK. A new look at IgE beyond allergies. F1000Res. (2019) 8:736. doi: 10.12688/f1000research.18186.1, PMID: 31168357 PMC6537913

[3] Ferastraoaru D Jordakieva G Jensen-Jarolim E. The other side of the coin: IgE deficiency, a susceptibility factor for Malignancy occurrence. World Allergy Organ J. (2021) 14:100505. doi: 10.1016/j.waojou.2020.100505, PMID: 33664932 PMC7887422

[4] Ferastraoaru D Zeig-Owens R Goldfarb DG Mueller AK Hall CB Weiden MD. Relationship between low serum immunoglobulin E levels and Malignancies in 9/11 World Trade Center responders. Ann Allergy Asthma Immunol. (2022) 129:769–75. doi: 10.1016/j.anai.2022.07.012, PMID: 35872243

[5] Weller KN McDonnell JC Albert JM Singer ME Hsieh FH. Increased hazard risk of first Malignancy in adults with undetectable serum IgE: a retrospective cohort study. J Clin Immunol. (2023) 43:568–77. doi: 10.1007/s10875-022-01401-7, PMID: 36380194

[6] McCraw AJ Chauhan J Bax HJ Stavraka C Osborn G Grandits M. Insights from IgE immune surveillance in allergy and cancer for anti-tumour IgE treatments. Cancers. (2021) 13:4460. doi: 10.3390/cancers13174460, PMID: 34503270 PMC8431713

[7] Sutton BJ Davies AM Bax HJ Karagiannis SN. IgE antibodies: from structure to function and clinical translation. Antibodies (Basel). (2019) 8:19. doi: 10.3390/antib8010019, PMID: 31544825 PMC6640697

[8] Pellizzari G Hoskin C Crescioli S Mele S Gotovina J Chiaruttini G. IgE re-programs alternatively-activated human macrophages towards pro-inflammatory anti-tumoural states. EBioMedicine. (2019) 43:67–81. doi: 10.1016/j.ebiom.2019.03.080, PMID: 30956175 PMC6562024

[9] Chauhan J McCraw AJ Nakamura M Osborn G Sow HS Cox VF. IgE antibodies against cancer: efficacy and safety. Antibodies (Basel). (2020) 9:55. doi: 10.3390/antib9040055, PMID: 33081206 PMC7709114

[10] Spicer J Basu B Montes A Banerji U Kristeleit R Miller R. Safety and anti-tumour activity of the IgE antibody MOv18 in patients with advanced solid tumours expressing folate receptor-alpha: a phase I trial. Nat Commun. (2023) 14:4180. doi: 10.1038/s41467-023-39679-9, PMID: 37491373 PMC10368744

[11] Spiegelberg HL. Structure and Function of Fc Receptors for IgE on Lymphocytes, Monocytes, and Macrophages. Adv Immunol. (1984). 35:61–88. doi: 10.1016/s0065-2776(08)60574-x, PMID: 6431765

[12] McDonnell JM Dhaliwal B Sutton BJ Gould HJ. IgE, IgE receptors and anti-IgE biologics: protein structures and mechanisms of action. Annu Rev Immunol. (2023) 41:255–75. doi: 10.1146/annurev-immunol-061020-053712, PMID: 36737596

[13] Santillan AA Camargo CA Colditz GA. A meta-analysis of asthma and risk of lung cancer (United States). Cancer Causes Control. (2003) 14:327–34. doi: 10.1023/a:1023982402137, PMID: 12846363

[14] Rosenberger A Bickeböller H McCormack V Brenner DR Duell EJ Tjønneland A. Asthma and lung cancer risk: a systematic investigation by the International Lung Cancer Consortium. Carcinogenesis. (2012) 33:587–97. doi: 10.1093/carcin/bgr307, PMID: 22198214 PMC3291861

[15] Fane M Weeraratna AT. How the ageing microenvironment influences tumour progression. Nat Rev Cancer. (2020) 20:89–106. doi: 10.1038/s41568-019-0222-9, PMID: 31836838 PMC7377404

[16] De Amici M Ciprandi G. The age impact on serum total and allergen-specific IgE. Allergy Asthma Immunol Res. (2013) 5:170–4. doi: 10.4168/aair.2013.5.3.170, PMID: 23638316 PMC3636452

[17] Hecht SS. Tobacco smoke carcinogens and lung cancer. J Natl Cancer Inst. (1999) 91:1194–210. doi: 10.1093/jnci/91.14.1194, PMID: 10413421

[18] Li C Lei S Ding L Xu Y Wu X Wang H. Global burden and trends of lung cancer incidence and mortality. Chin Med J (Engl). (2023) 136:1583–90. doi: 10.1097/cm9.0000000000002529, PMID: 37027426 PMC10325747

[19] O’Keeffe LM Taylor G Huxley RR Mitchell P Woodward M Peters SAE. Smoking as a risk factor for lung cancer in women and men: a systematic review and meta-analysis. BMJ Open. (2018) 8:e021611. doi: 10.1136/bmjopen-2018-021611, PMID: 30287668 PMC6194454

[20] Alvela-Suarez L Campos J Carballo I Gomez-Rial J Vidal C Lombardero M. False-positive results of serological tests for allergy in alcoholic patients. J Investig Allergol Clin Immunol. (2019) 29:213–21. doi: 10.18176/jiaci.0309, PMID: 30183656

[21] Ahmed NJ Husen AZ Khoshnaw N Getta HA Hussein ZS Yassin AK. The effects of smoking on IgE, oxidative stress and haemoglobin concentration. Asian Pac J Cancer Prev. (2020) 21:1069–72. doi: 10.31557/apjcp.2020.21.4.1069, PMID: 32334472 PMC7445955

[22] Linos E Raine T Alonso A Michaud D. Atopy and risk of brain tumors: a meta-analysis. J Natl Cancer Inst. (2007) 99:1544–50. doi: 10.1093/jnci/djm170, PMID: 17925535

[23] Helby J Bojesen SE Nielsen SF Nordestgaard BG. IgE and risk of cancer in 37–747 individuals from the general population. Ann Oncol. (2015) 26:1784–90. doi: 10.1093/annonc/mdv231, PMID: 25969367

[24] Guerra G Nakase T Kachuri L McCoy L Hansen HM Rice T. Association of immunoglobulin E levels with glioma risk and survival. J Natl Cancer Inst. (2025) 117:545–53. doi: 10.1093/jnci/djae265, PMID: 39447063 PMC11884848

[25] Radzikowska U Baerenfaller K Cornejo-Garcia JA Karaaslan C Barletta E Sarac BE. Omics technologies in allergy and asthma research: an EAACI position paper. Allergy. (2022) 77:2888–908. doi: 10.1111/all.15412, PMID: 35713644 PMC9796060

[26] Lang C Egger F Hoda MA Querner AS Ferencz B Lungu V. Lymphocyte-to-monocyte ratio is an independent prognostic factor in surgically treated small cell lung cancer: an international multicenter analysis. Lung Cancer. (2022) 169:40–6. doi: 10.1016/j.lungcan.2022.05.010, PMID: 35643059

[27] Wu Y Yuan M Wang C Chen Y Zhang Y Zhang J. T lymphocyte cell: a pivotal player in lung cancer. Front Immunol. (2023) 14:1102778. doi: 10.3389/fimmu.2023.1102778, PMID: 36776832 PMC9911803

[28] Ugel S Canè S Sanctis FD Bronte V. Monocytes in the tumor microenvironment. Annu Rev Pathol Mech Dis. (2021) 16:93–122. doi: 10.1146/annurev-pathmechdis-012418-013058, PMID: 33497262

[29] Vukovic N Halabi S Russo-Cabrera JS Blokhuis B Berraondo P Redegeld FA. A human IgE bispecific antibody shows potent cytotoxic capacity mediated by monocytes. J Biol Chem. (2022) 298:102153. doi: 10.1016/j.jbc.2022.102153, PMID: 35718062 PMC9293656

[30] Ezeamuzie CI Al-Attiyah R Shihab PK Al-Radwan R. Low-affinity IgE receptor (FcϵRII)-mediated activation of human monocytes by both monomeric IgE and IgE/anti-IgE immune complex. Int Immunopharmacol. (2009) 9:1110–4. doi: 10.1016/j.intimp.2009.05.009, PMID: 19505590

[31] Jensen-Jarolim E Bax HJ Bianchini R Capron M Corrigan C Castells M. AllergoOncology – the impact of allergy in oncology: EAACI position paper. Allergy. (2017) 72:866–87. doi: 10.1111/all.13119, PMID: 28032353 PMC5498751

